# Plant Phenology and Absence of Sex-Biased Gall Attack on Three Species of *Baccharis*

**DOI:** 10.1371/journal.pone.0046896

**Published:** 2012-10-04

**Authors:** Mário M. Espírito-Santo, Frederico S. Neves, G. Wilson Fernandes, Jhonathan O. Silva

**Affiliations:** 1 Departamento de Biologia Geral, Centro de Ciências Biológicas e da Saúde, Universidade Estadual de Montes Claros, Montes Claros, Minas Gerais, Brazil; 2 Ecologia Evolutiva & Biodiversidade/DBG. ICB/Universidade Federal de Minas Gerais, Belo Horizonte, Minas Gerais, Brazil; 3 Departamento de Ecologia, Instituto de Ciências Biológicas, Universidade de Brasília, Brasília, Distrito Federal, Brazil; Jyväskylä University, Finland

## Abstract

**Background:**

Dioecy represents a source of variation in plant quality to herbivores due to sexual differences in intensity and timing of resource allocation to growth, defense and reproduction. Male plants have higher growth rates and should be more susceptible to herbivores than females, due to a lower investment in defense and reproduction.

**Methodology/Principal Findings:**

We compared resource investment to growth and reproduction and its consequences to herbivore attack on three *Baccharis* species along one year (*B. dracunculifolia*, *B. ramosissima,* and *B. concinna*). Phenological patterns presented by the three species of *Baccharis* were quite different over time, but the number of fourth-level shoots and plant growth rate did not differ between sexes in any studied species. Intersexual difference in reproductive investment was only observed for *B. concinna*, with female individuals supporting higher inflorescence density than male individuals throughout the year. Gall abundance on the three *Baccharis* species was not influenced by plant sex. However, all plant traits evaluated here positively influenced the gall abundance on *B. concinna*, whereas only the number of fourth-level shoots positively influenced gall abundance on *B. ramosissima* and *B. dracunculifolia*.

**Conclusions/Significance:**

The absence of differential reproductive allocation may have contributed to similar growth and shoot production between the sexes, with bottom-up effects resulting in gender similarities in gall abundance patterns. The number of fourth-level shoots, an indicator of meristem availability to herbivores, was the most important driver of the abundance of the galling insects regardless of host plant gender or species. Albeit the absence of intersexual variation in insect gall abundance is uncommon in the literature, the detailed study of the exceptions may bring more light to understand the mechanisms and processes behind such trend.

## Introduction

Plants have limited resources to invest in growth, reproduction and defense against herbivores [1]. In general, resource demand for these three processes cannot be held simultaneously, due to a tradeoff in resource allocation among these different physiological processes [1]–[3]. Differential resource allocation produces changes in plant traits (e.g. architecture, growth, concentration of carbon-based defensive compounds), that can extend its effects on upper trophic levels [2], [4], [5]. Fast-growing species or sexes (within dioecious species; e.g., male plants) are expected to invest less in chemical and/or structural defenses [1]. On the other hand, their counterparts usually invest a higher amount of nutrients in reproduction, and the consequent decrease in nitrogen levels limits the production of new leaves. Thus, female plants or slow-growing species are expected to protect their limited growth potential against tissue loss via herbivory [1], [4], [6].

Plant sex was explored as a source of variation in quality to herbivores in many studies with dioecious species. In general, due to intersexual differences in resource allocation, male plants are more susceptible to herbivore attack than females (the sex-biased herbivory hypothesis) [7]–[11]. Several studies conducted mainly in temperate regions have detected higher herbivory levels on male plants. However, a male-biased herbivore attack has not been corroborated in some recent studies in tropical systems involving *Baccharis* shrubs [9], [12], [13]. *Baccharis* (Asteraceae) is a genus with over 500 species distributed from United States to Argentina. All species are perennial and dioecious shrubs, except for *Baccharis monoica* [14]. *Baccharis* likely supports the richest galling-inducing insects fauna studied so far in the Neotropics, with 121 galls species on 40 host plant species [15]. With such a rich community in which the gall-inducing insects belong to several different taxa, the system comprised of *Baccharis* species and their gall-inducing insects provides an ideal scenario to test for plant sexual differences on intensity and timing of resource allocation and herbivory attack.

Gall-inducing insects are highly specialized to their host and each species attacks only a single plant organ [16]–[18]. Galling insects are able to manipulate plant chemical defenses [19], [20], but are strongly influenced by the production of new tissues (meristems) and/or faster growth or vigorous modules of their hosts [21]–[25]. Thus, attack by these insects can be synchronized to periods in which there is an increased production of highly nutritious young tissues or fast plant growth [26]–[27]. Due to their finely-tuned interaction with the host plants, galling insects provide excellent opportunities to evaluate the influence of bottom-up forces on herbivores [28], [29]. In a recent meta-analysis on gender effects on plant-herbivore interactions, Cornelissen & Stiling [8] highlighted the prevalence of galling insects as subjects of sex-biased herbivory studies, representing almost 35% of the independent comparisons reviewed. Moreover, these authors found that the abundances of folivores and galling insects were more strongly influenced by plant sex than other feeding guilds, and intersexual differences in plant morphological, phenological and/or nutritional characteristics might be responsible for this result.

Plant vegetative and reproductive phenology can be influenced by several abiotic (water availability, photoperiod and wind speed, among others) and biotic (presence of herbivores, pollinators and seed dispersers) factors [30]–[32]. In dioecious species, phenology is related to patterns of vegetative and sexual development [7]–[9], [33]–[36]. Furthermore, trade-offs in biomass allocation between reproduction and growth can lead to temporal variations in vegetative investment in both plant sexes, affecting resource availability for their associated herbivores [8], [36], [37]. Male plants usually present a higher investment in flower production early in the reproductive season, whereas females divert most of their energy to sustain fruit maturation later in the reproductive season [33], [38]. If a trade-off between growth and reproduction occurs, it is expected that males would suffer higher galling attack later in the reproductive season, the contrary being true for female plants. In this way, studies regarding plant intersexual differences and herbivory should consider resource investment across the entire growing and reproductive seasons due to the possible contrasting phenologies between male and female hosts (e.g., [10], [33], [35], [39]).

This study aimed to explore the relationships between galling insect attack and dioecy on three *Baccharis* species, mediated by investment in number of shoots, relative shoot growth rate and number of inflorescences. We assessed resource allocation to reproduction and growth between male and female plants along one year. The following questions were asked: i) Does resource allocation to reproductive and vegetative functions differ between male and female plants and, if so, does differential resource allocation provoke differences in gall attack between sexes? ii) Are there temporal differences in vegetative and reproductive investment between sexes and, if so, how such variations affect gall abundance across time?

## Materials and Methods

### Study System

*Baccharis dracunculifolia* DC is a widespread dioecious perennial shrub ranging from 2 to 3 m in height and is found in Brazil, Argentina, Uruguay, Paraguay and Bolivia [40]. *Baccharis ramosissima* Gardner reaches from 2 to 4 m in height and occurs predominantly in Minas Gerais state, Brazil [40]. Finally, *Baccharis concinna* Barroso is usually 1–2 m tall and is endemic to Serra do Cipó, Minas Gerais, Brazil [40]. Fernandes et al. [15] recorded 17, 15, and 11 different gall-inducing species on *B. dracunculifolia*, *B. concinna,* and *B. ramosissima*, respectively. Each *Baccharis* species support a monophagous species of gall-inducing *Baccharopelma* [41]. *Baccharopelma dracunculiafoliae* Burckhardt is the most common galling herbivore found on *B. dracunculifolia*, whereas *Baccharopelma concinnae* Burckhardt dominates the galling abundance on *B. concinna*, the same occurring for *Baccharopelma brasiliensis* Burckhardt on *B. ramosissima* (see [15], [41] for details).

### Study Sites

The populations of *B. dracunculifolia* and *B. ramosissima* were located in the Campus of the Universidade Federal de Minas Gerais, Belo Horizonte, Brazil (19° 30′ S, 44° 00′ W), at 805 m above sea level. The average temperature of the study site varies from 18°C to 20°C, and the average annual precipitation is 1,500 mm [12]. The vegetation is extremely heterogeneous and disturbed, composed of native, introduced, ornamental and fruit-bearing species. The adjacent native vegetation contains dry forest and cerrado (savanna) species [42]. The plants were all located in a 3 ha area at an early successional stage, dominated by *B. dracunculifolia*, *B. ramosissima*, grasses and herbaceous and shrubby leguminous species [43].

The population of *B. concinna* was situated at Serra do Cipó, in the Espinhaço Mountain Chain, approximately 100 km from Belo Horizonte. This region is characterized by quartzitic soils covered by rupestrian fields, highly xerophytic vegetation with predominance of herbs and shrubs [44]. The study site was located at 1,250 m above the sea level (19° 17′ S, 43° 35′ W), inside a private protected area (Reserva Particular Vellozia). The climate is similar to the observed in Belo Horizonte, with average annual precipitation of approximately 1,500 mm and average temperature between 17.4–19.8°C [45].

**Figure 1:**
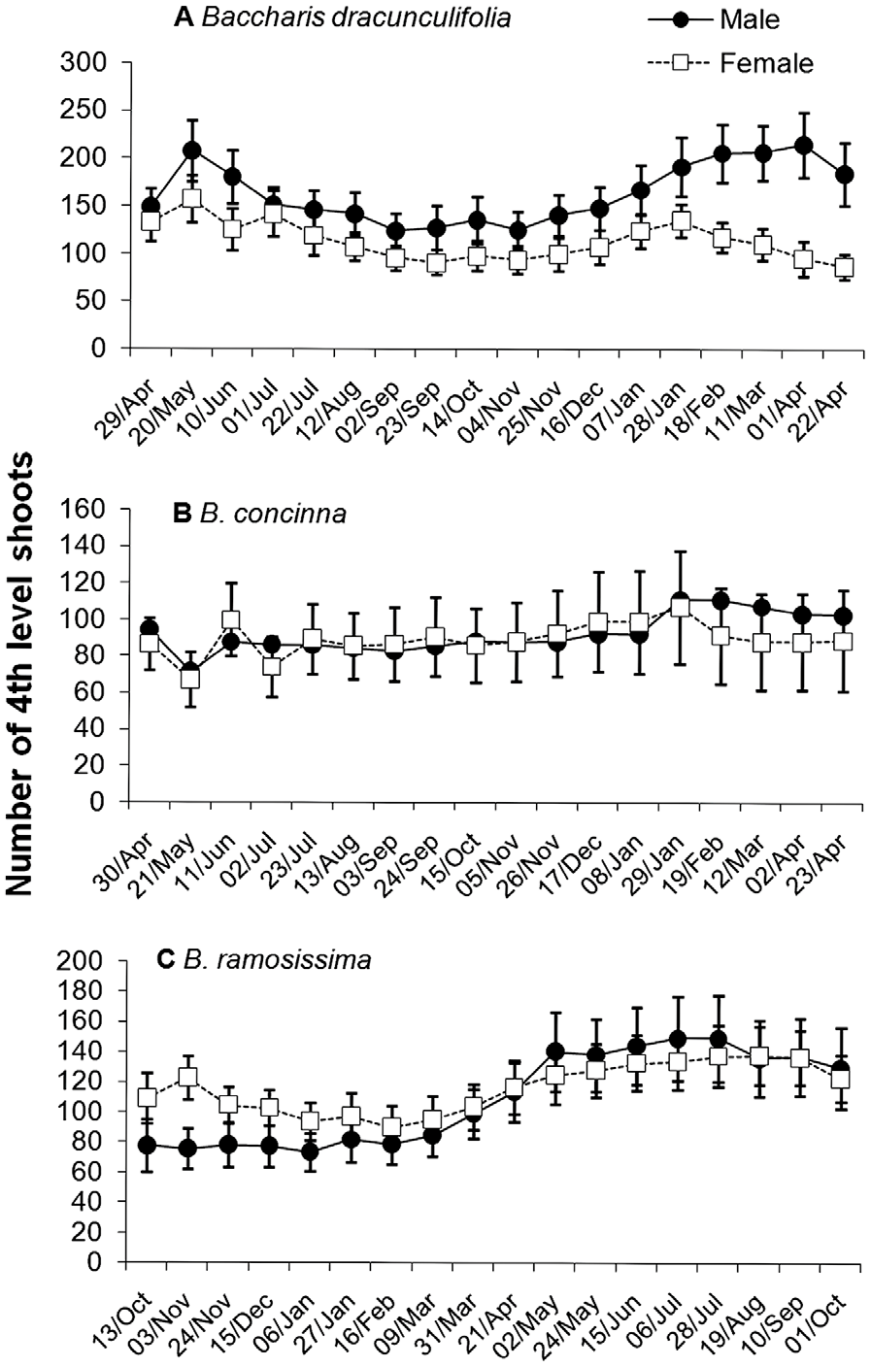
Average number of fourth-level shoots (mean ± s.e.) in male and female individuals of (a) *Baccharis dracunculifolia*, (b) *B. concinna* and (c) *B. ramosissima*. Fourth-level shoots were counted every three weeks during one year.

**Table 1:** ANOVA of the minimal linear mixed-effects (LME) models to evaluate the effects of plant sex on the number of shoots, relative growth rate and inflorescence density in three species of *Baccharis*.

	*B. dracunculifolia*			*B. concinna*			*B. ramosissima*		
Source	Df	F	p	df	F	p	df	F	P
Number of shoots									
Intercept	1, 479	2478.9	<0.0001	1, 479	2886.4	<0.0001	1, 478	1395.7	<0.0001
Date			ns			ns	1, 478	14.0	<0.0005
Plant sex			ns			ns			ns
Date×Plant sex			ns			ns			ns
Relative shoot growth rate									
Intercept	1, 479	589.3	<0.0001	1, 478	143.1	<0.0001	1, 478	239.3	<0.0001
Date			ns	1, 478	5.32	<0.05	1, 478	72.8	<0.0001
Plant sex			ns			ns			ns
Date×Plant sex			ns			ns			ns
Inflorescence density									
Intercept	1, 477	55.0	<0.0001	1, 477	416.1	<0.0001	1, 478	38.7	<0.0001
Date	1, 477	107.8	<0.0001			ns	1, 478	83.5	<0.0001
Plant sex			ns	1, 28	29.4	<0.0001			ns
Date×Plant sex	1, 477	4.22	<0.05	1, 477	4.31	<0.05			ns

**Figure 2:**
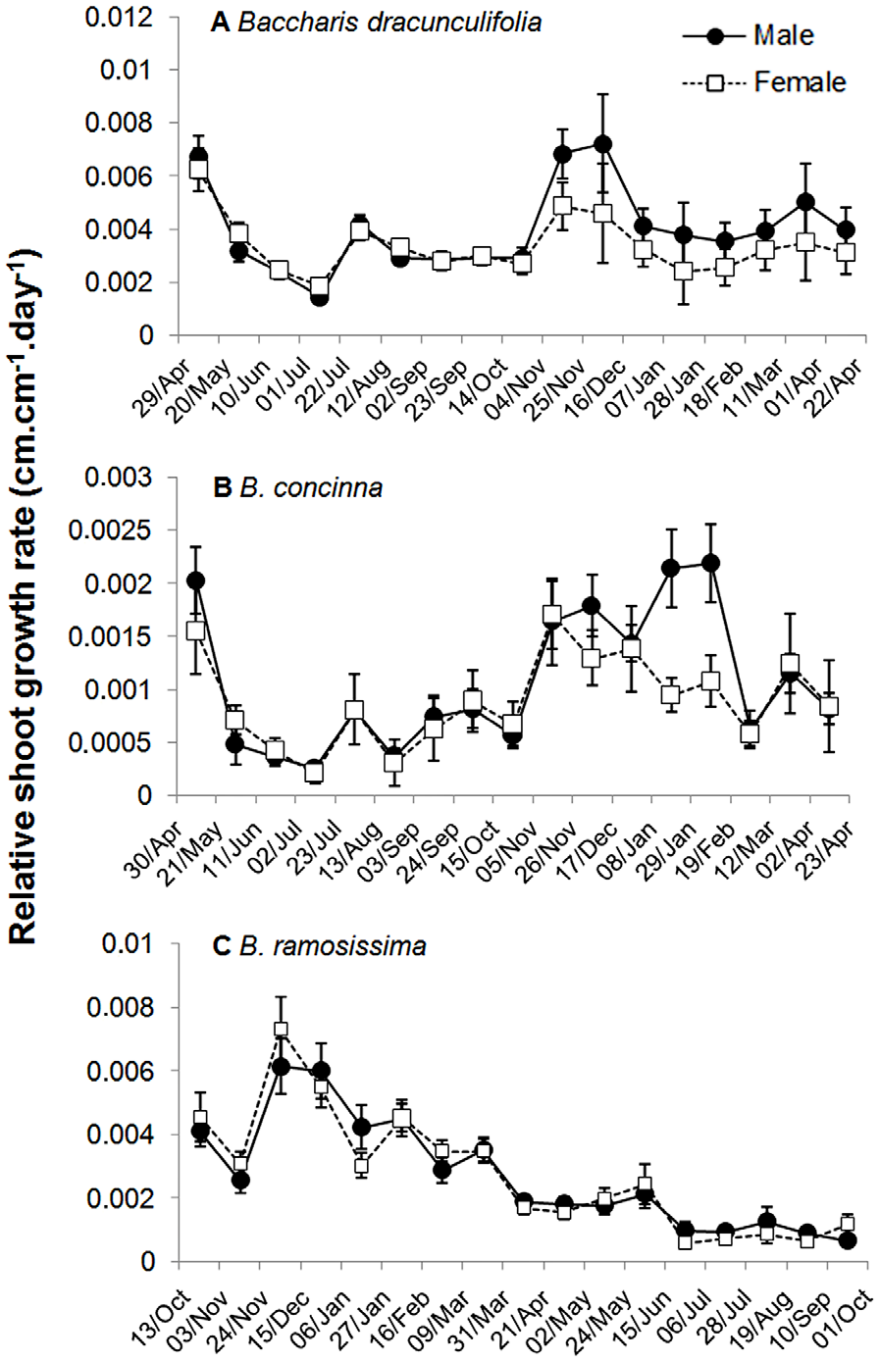
Average shoot relative growth rates (cm.cm^−1^.day^−1^) in male and female individuals of (a) *Baccharis dracunculifolia*, (b) *B. concinna* and (c) *B. ramosissima*. Shoot measurements were performed every three weeks during one year (mean ± s.e.).

**Figure 3:**
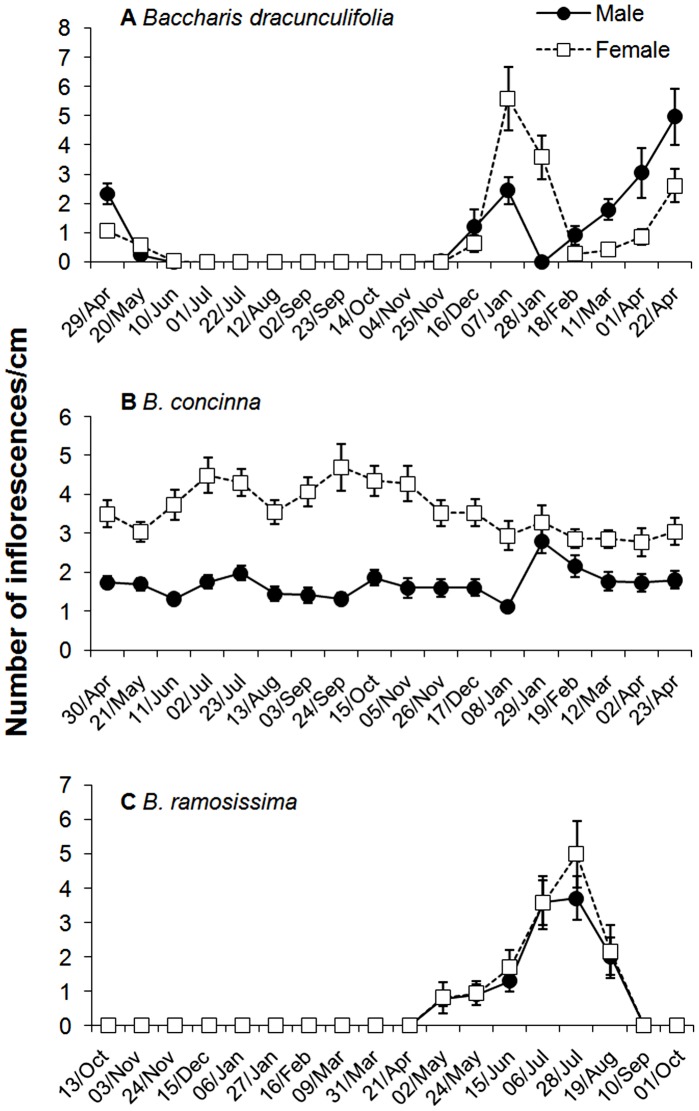
Average density of inflorescences (mean ± s.e.) in male and female individuals of (a) *Baccharis dracunculifolia*, (b) *B. concinna* and (c) *B. ramosissima*. Inflorescences were counted every three weeks during one year.

### Samples

Thirty individuals (15 male and 15 female) of each *Baccharis* species were arbitrarily marked in field. Plant sex was determined by analysis of floral morphology (see [22] for details). We arbitrarily selected three secondary branches on each plant, which were tagged with thin, colored adhesive tape placed on their terminal 15 cm, and shoot length was measured every three weeks. Shoot relative growth rate was calculated as: [(Log_e_ final length – Log_e_ initial length)/(time final-time initial)] for each measurement date. Total number of inflorescences was counted for all marked shoots and inflorescence density was calculated by shoot centimeter. Both shoot growth and inflorescence densities were averaged per plant for statistical analyses. The number of fourth-level shoots was counted for each individual at the same intervals during one year (totaling 18 repeated measures). Fourth-level shoots are usually young and possess many sprouting leaves, thus this variable may be considered as an indication of the amount of active meristems in a given individual. Higher-level shoots (e.g., fifth and sixth level) are encountered only in few architecturally complex individuals and it would not be helpful to indicate meristem number for most of the plants sampled in this study [22]. In *B. dracunculifolia* and *B. concinna,* phenological changes were recorded from May 2001 to May 2002, while in *B. ramosissima,* phenology was recorded from October 2001 to October 2002. In each sampling date, all live galls found on plants were counted without removal or marking. Thus, the same galls could have been occasionally counted during successive samplings. Galls were considered alive when exit holes from either the gall-inducer or parasitoids were absent from external walls. The present study considered only insect-induced galls [15].

### Analysis

To examine the intersexual differences in the number of fourth-level shoot, relative growth rate and inflorescence density along one year, we adjusted linear mixed-effect models (LME) for each of these response variables for each *Baccharis* species [46]. These analyses were employed because the data were obtained repeatedly in the same plants during subsequent intervals, and the temporal autocorrelation generated by consecutive counting violates the assumption of sampling independence. Assuming independence when it is not true would inflate the error degrees of freedom and could lead to spurious significance (Type I error) [46]. To overcome this problem, the data were grouped by plant and the error variances were calculated for each different group. In this case, the response is not the individual measure, but the sequence of measures in an individual [46]. Plant sex, date and the interaction between sex and date were used as explanatory variables (fixed effects), whereas the resulting groups per plant were treated as random effects (date by plant identity).

To verify the effects of the date, sex and plant traits (number of fourth-level shoot, relative growth rate and inflorescence density) on gall abundance on the three *Baccharis* species, LMEs were also adjusted as described for the previous models. For these models, the response variables were log-transformed to meet normality. Minimal adequate models were adjusted with the removal of the non-significant terms. All model and analyses were conducted using the software R_2.11_ [47]. All data are given as average ± standard error.

## Results

### Resource Allocation to Growth and Reproduction

The phenological patterns exhibited by the three species of *Baccharis* were quite different (Figures 1, 2, 3). The number of fourth-level shoots varied significantly during the study period only for *B. ramosissima,* increasing after the end of the rainy season in March. Shoot number did not differ between male and female plants for all three species (Table 1, Figures. 1a, b, c). Although no significant temporal differences were observed for *B. dracunculifolia*, there was a peak on shoot growth for this species in November-December, at the beginning of the rainy season (Figure 2a). On the other hand, relative shoot growth rate varied significantly along the year for *B. concinna* and *B. ramosissima*. In *B.concinna*, higher growth occurred from November to February (rainy season) declining in March, with an additional peak in May (at the beginning of the dry season). The lowest growth rate was observed between July and October (dry season, Figure 2b). For *B. ramosissima*, a similar pattern was verified: higher shoot growth rate was observed during the rainy season (November-February), followed by a steady decline in March reaching almost zero between July and August (dry season, Figure 2c). Shoot growth rates were statistically similar between sexes for all species during this study (Table 1, Figures 2a, b, c).

The pattern of inflorescence production showed marked contrasts between the three *Baccharis* species (Figures 3 a, b, c). Inflorescence density varied significantly along the year for *Baccharis dracunculifolia* (Table 1), with two peaks of flowering: in the middle of the rainy season (December-January) and in the beginning of the dry season (April-May). Although the total inflorescence density did not differ between sexes, the temporal pattern of reproductive investment was sexually distinct for this species. In the first flowering peak, we observed a higher inflorescence density for the female gender, whereas in the second peak the opposite trend was detected (Figure 3a). *Baccharis concinna* presented inflorescences during the entire year, but the temporal pattern differed between sexes. For female plants, inflorescence density was slightly higher during the dry season, whereas males showed a peak in inflorescence production in the middle of the rainy season in January. As a whole, inflorescence density was constantly higher in female plants in this species (Table 1, Figure 3b). On the other hand, inflorescence density in *B. ramosissima* varied greatly during the year but did not differ between sexes (Table 1). For this species, inflorescences were produced only once a year, in the middle of the dry season (April-September) (Figure 3c). In general, the only plant trait considered in the present study that differed between sexes was the inflorescence density in *B. concinna.*

### Sex-mediated Herbivore Attack

*Baccharis dracunculifolia* supported the highest richness (14 species) and abundance (20,327 counts) of insect galls among the studied host species. Both *B. concinna* and *B. ramosissima* showed 10 galling morphospecies each. *Baccharis concinna* individuals presented a total abundance of 8,353 insect galls whereas *B. ramosissima* supported 5,410 insect galls during the study period. Most of the galls were induced by psyllids belonging to the genus *Baccharopelma* (Hemiptera: Psyllidae). *Baccharopelma dracunculiafoliae* induced 80% of the galls on *B. dracunculifolia*, whereas *Baccharopelma concinnae* induced 65% of the galls on *B. concinna*, the same occurring for *Baccharopelma brasiliensis* on *B. ramosissima* (70% of the galls). The galls induced by the other insects had a very low relative abundance on the three *Baccharis* species.

**Figure 4:**
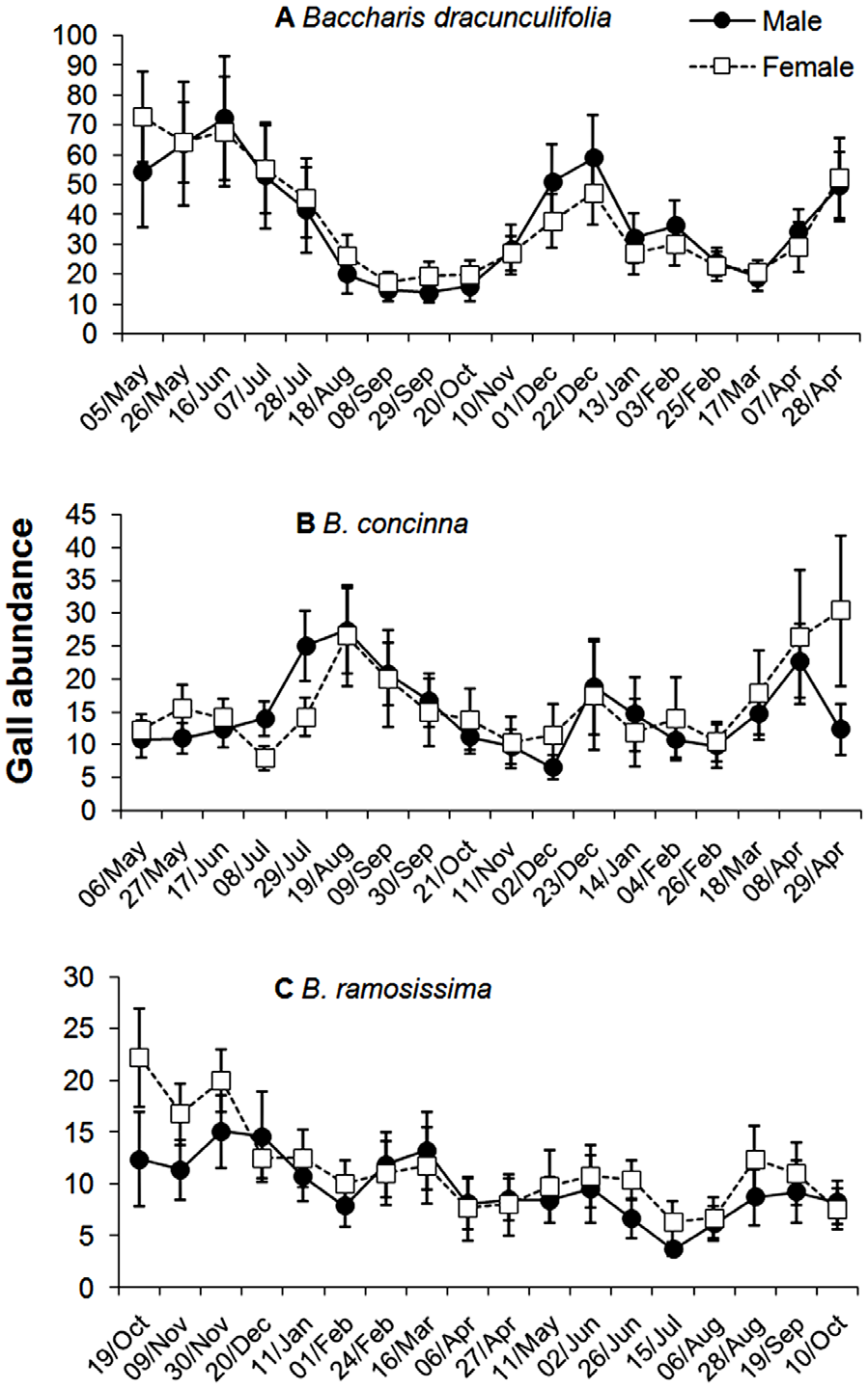
Average number of galls (mean ± s.e.) on male and female individuals of (a) *Baccharis dracunculifolia*, (b) *B. concinna* and (c) *B. ramosissima*. Galls were counted every three weeks during one year.

Gall abundance showed different patterns of temporal variation for each *Baccharis* species (Table 2, Figure 4). Although no statistically significant difference in gall abundance was detected on *B. dracunculifolia* along time (Table 2), a bimodal pattern of attack was observed, with peaks in the transition between the rainy and dry season (April-June) and in the beginning of the rainy season (December) (Figure 4a). For *B. concinna,* gall abundance varied significantly along time (Table 2) and three peaks were recorded: in the middle of the dry season (July-August 2001), at the beginning (December 2001), and at the end (April 2002) of the rainy season. Significant temporal variations were also recorded for *B. ramosissima*, and the highest abundance of insect galls was observed in the rainy season (October-January) (Figure 4c).

**Table 2:** ANOVA of the minimal linear mixed-effects (LME) models to evaluate the effects of date, sex and plant traits on gall abundance in three species of *Baccharis*. Non-significant terms were deleted from complete models through stepwise removal.

	*B. dracunculifolia*			*B. concinna*			*B. ramosissima*		
Source	df	F	p	Df	F	P	df	F	P
Intercept	1, 476	502.71	<0.0001	1, 476	434.8	<0.0001	1, 478	274.0	<0.0001
Date			ns	1, 476	7.39	<0.01	1, 478	20.5	<0.0001
Plant sex			ns			ns			ns
Date×Plant sex			ns			ns			ns
Number of shoots	1, 476	72.3	<0.0001	1, 476	21.6	<0.0001	1, 478	5.84	<0.05
Relative shoot growth rate	1, 476	13.4	<0.0001	1, 476	6.15	<0.05			ns
Inflorescence density			ns	1, 476	7.23	<0.01			ns

Gall abundance did not differ between sexes in any of the three species of *Baccharis* (Table 2, Figure 4), not even in *B. concinna* which presented strong intersexual differences in inflorescence production. Also, the temporal pattern of gall attack was quite similar between male and female individuals in all host species during the entire study period (Figure 4). Gall abundance correlated positively with the number of fourth-level shoots on the three species of *Baccharis* (Table 2). Relative shoot growth rate also influenced positively the gall number on *B. dracunculifolia* and *B. concinna* (Table 2). Indeed, a higher gall abundance was recorded in the periods of greatest relative growth for all host species, although no significant relationship was detected in *B. ramosissima* (Table 2, Figures 2 and 4). Inflorescence density only affected positively the gall abundance on *B. concinna*, but this effect did not differ between sexes.

## Discussion

### Resource Allocation to Growth and Reproduction

Plant sex does not seem to be an important source of variation in phenology and gall attack on *Baccharis*, corroborating previous studies in this speciose genus [9], [12], [13], [33], [48], [49]. In spite of some intersexual differences in inflorescence production patterns in *B. dracunculifolia* and *B. concinna*, shoot number and growth rates were similar between genders in all three species. Thus, we found little support for the existence of differential resource allocation to growth and reproduction between sexes. In dioecious species, male and female individuals have different reproductive functions (i.e., pollen donation vs. seed production and fruit maturation) and, in many cases, fleshy fruits and nutrient-rich seed structures are energy-costly [38], [50]–[52], thus leading to strong asymmetries in reproductive investment between sexes. In *Baccharis*, inflorescences are small and comparable in size between male and female individuals of all three species, and seeds are tiny and wind-dispersed [53]. Thus, female plants probably do not have a reproductive investment greater enough than males to produce differences in meristem number and plant growth.

In the case of *B. concinna*, females indeed produced a significantly higher number of inflorescences than males. However, this species is wind-pollinated [40] and has the smallest inflorescences among the three species studied here. This differential investment did not affect plant growth and shoot production, which remained roughly similar for both sexes during the entire year, suggesting the absence of trade-offs in this species (i.e., male *B. concinna* individuals had both highest growth rate and inflorescence density early in the rainy season, in December-February). Similarly, Antos and Allen [52] did not find a significant correlation between reproductive effort and growth in either sex of the shrub *Oemleria cerasiformis* (Rosaceae). Temporal variations in inflorescence production may represent a response to other factors that regulate reproductive investment (e.g. availability of light and soil nutrients and moisture) [38], [50], [52]. Thus, the small wind-pollinated inflorescences of *B. concinna* are likely relatively inexpensive in terms of resource allocation and affordable throughout the year for both genders.

For *B. dracunculifolia*, the timing of reproduction was different between sexes. Although male plants started reproduction earlier, females bloomed in January, in the peak of the rainy season, and this massive investment probably limited their inflorescence production for the rest of the season. The low reproductive male investment in January may have been caused by their higher resource allocation to growth in the previous month, saving energy for inflorescence production later in the season (see Figure 2a). The pattern detected here was opposite to the observed by Espírito-Santo et al. [33], who reported a higher male investment early in the season related to pollinator attraction, and a late female reproductive investment due to seed maturation (especially in June). However, this previous study was much more limited in time (March-June), and there were strong inter-annual variations in reproductive investment of *B. dracunculifolia* (i.e., no inflorescences in June in the present study). Thus, our results suggest that sexual differences in phenology may not be determined only by male and female reproductive functions (pollen donation vs. seed production and fruit maturation), and are also affected by abiotic (e.g., water availability) and biotic (e.g., interspecific competition for pollinators) pressures. In this way, long-term studies are necessary to detect temporal patterns in reproductive investment in dioecious plants.

For the studied *Baccharis* species, the majority of changes in phenological events was not related to plant sex, but varied between species and is likely associated with resource availability in each plant population. In *B. dracunculifolia* and *B. concinna*, the higher number of fourth-level shoots and relative shoot growth rate occurred during the rainy season. In contrast, *B. ramosissima* produced new branches and flowers in the dry season, when shoot growth rate was reduced. Thus, this species may have not been able to sustain growth and reproduction simultaneously, especially in periods of resource scarcity. In *B. ramosissima*, inflorescences of both sexes are similar in size and pollination is performed by social bees [40]. Blooming in the middle of the dry season is not unusual in some tropical environments, since the low air humidity contributes to increase nectar concentration and leaf fall may improve flower visibility by pollinators [54]. In the case of *B. ramosissima*, it is likely that this strategy reduces competition for pollinators with the sympatric *B. dracunculifolia*. These two species are very similar in architecture [22] and in inflorescence morphology. Since *B. ramosissima* populations are usually much less dense when co-occurring with *B. dracunculifolia* (M. M. Espírito-Santo, personal observation), it is likely that there was a strong evolutionary pressure for niche differentiation causing both sexes of *B. ramosissima* to reproduce very synchronously during periods of water scarcity but high pollinator availability.

### Sex-mediated Herbivore Attack

The gall abundance on the studied species of *Baccharis* did not conform to the pattern predicted by the sex-biased herbivory hypothesis. On the other hand, this result corroborates several other studies related to this genus in Brazil [9], [12], [13], [49], [55]. Furthermore, the present study involved multiple hosts and galling species, and closely followed temporal changes in gall abundance and host resource availability along one year. Therefore, we have consistent support to reject the male-biased attack prediction of the sex-biased herbivory hypothesis.

The absence of the gender-related gall attack is explained by the lack of differences in plant growth rate and meristem availability (estimated indirectly by the number of fourth-level shoots) between male and female plants. Indeed, meristem number was already reported as the most important plant trait driving gall abundance and species richness across several species of *Baccharis* [22]. This assertion is reinforced by the positive relationship between gall abundance and shoot number and plant growth rate in the present study. However, other plant traits not evaluated here such as nutrient content, secondary chemistry and physical defenses can differ between sexes and affect gall attack (see [8]). Although we did not quantify plant defenses, there is some evidence that carbon-based compounds such as tannins are not abundant and do not vary between sexes in *B. dracunculifolia* [12], [48], but nothing is yet known for *B. concinna* and *B. ramosissima*. Furthermore, gall-inducing insects are capable of manipulating chemical composition, growth rates and developmental patterns of the attacked plant organ [19], [20], [56], and are able to overcome chemical and physical defenses regardless of plant gender. Thus, the similarities in gall abundance between male and female individuals in this host genus are likely related to plant vigor and availability of young tissues for gall induction, but leaf nutrients and defenses deserve further investigation.

Gall abundance was influenced by plant phenology, being mainly affected by temporal variations in shoot growth rates. Indeed, gall attack was higher mostly during the rainy season, when shoot growth was more vigorous in all three species. Although the number of fourth-level shoots was the most important plant trait affecting gall abundance at the individual level, statistically significant temporal variations in this variable were only detected in *B. ramosissima.* However, shoot production in this species increased during the dry season and may be related to flowering, when shoot growth and gall attack were both low. For *B. dracunculifolia*, other studies detected an increase in the number of fourth-level shoots and galls in March-April, right before the second peak of flowering [12], [33]. However, such phenological pattern was only observed for male plants of this species in the present study, and a higher gall attack did not track shoot number. Thus, it seems that plant growth rates are more important in determining temporal variations in gall attack, whereas the number of fourth-level shoots is responsible for spatial, individual differences in plant susceptibility.

### Conclusion

Overall, the *Baccharis* species studied here did not exhibit sexual dimorphisms on vegetative traits that are usually related to distinct reproductive functions. The absence of differential reproductive allocation may have contributed to similar growth and shoot production between male and female plants. As a bottom-up consequence, plant gender is a weak predictor, or even an irrelevant variable influencing gall abundance in this host plant genus. In spite of that, the plant traits evaluated here are important drivers of gall attack both in space (between individuals) and time, and temporal changes in gall abundance are synchronized with resource availability (e.g. young tissues and vigorous growth) throughout the year. The absence of sex-biased herbivory can be more frequent than usually reported, since studies that fail to detect intersexual variation on herbivore attack in other system are probably underrepresented in the literature.

## References

[1] Herms DA, Mattson WJ (1992) The dilemma of plants: To grow or defend. Q Rev Biol 67: 283–335.

[2] Cornelissen TG, Fernandes GW (2001) Defence, growth and nutrient allocation in the tropical shrub *Bauhinia brevipes* (Leguminosae). Austral Ecol 26: 246–253.

[3] Weiner J (2004) Allocation, plasticity and allometry in plants. Perspect. Plant Ecol Evol. Syst. 6: 207–215.

[4] Bryant JP, Chapin III FS, Klein DR (1983) Carbon/nutrient balance of Boreal plants in relation to vertebrate herbivory. Oikos 40: 357–368.

[5] Imaji A, Seiwa K (2010) Carbon allocation to defense, storage and growth in seedlings of two temperate broad-leaved tree species. Oecologia 162: 361–369. 10.1007/s00442-009-1453-3 19763628

[6] Cepeda-Cornejo V, Dirzo R (2010) Sex-related differences in reproductive allocation, growth, defense and herbivory in three dioecious Neotropical palms. PloS One 5: e9824 doi: 10.1371/journal.pone.0009824. 20352113 10.1371/journal.pone.0009824 PMC2843723

[7] Ågren J (1987) Intersexual differences in phenology and damage by herbivores and pathogens in dioecious *Rubus chamaemorus* L. Oecologia. 72: 161–169. 10.1007/BF00379262 28311534

[8] Cornelissen T, Stiling P (2005) Sex-biased herbivory: A meta-analysis of the effects of gender on plant-herbivore interactions. Oikos 111: 488–500.

[9] Carneiro MAA, Fernandes GW, de Souza OFF, Souza WVM (2006) Sex-mediated herbivory by galling insects on *Baccharis concinna* (Asteraceae). Rev Bras Entomol 50: 394–398.

[10] Watson MA (1995) Sexual differences in plant development phenology affect plant-herbivore interactions. Trends Ecol Evol 10: 180–182. 21236996 10.1016/s0169-5347(00)89046-1

[11] Boecklen WJ, Price PW, Mopper S (1990) Sex and drugs and herbivores: sex-biased herbivory in arroyo willow (*Salix lasiolepis*). Ecology 71: 581–588.

[12] Espírito-Santo MM, Fernandes GW (1998) Abundance of *Neopelma baccharidis* (Homoptera: Psyllidae) galls on the dioecious shrub *Baccharis dracunculifolia* (Asteraceae). Environ Entomol 27: 870–876.

[13] Faria ML, Fernandes GW (2001) Vigour of a dioecious shrub and attack by a galling herbivore. Ecol Entomol 26: 36–45.

[14] Nesom G (1988) *Baccharis monoica* (Compositae: Asteraceae), a monoecious species of the *B. salicifolia* complex from Mexico and Central America. Phytologia 65: 160–164.

[15] Fernandes GW, Carneiro MAA, Lara ACF, Allain LA, Andrade GI, et al (1996) Galling insects on Neotropical species of *Baccharis* (Asteraceae). Trop Zool 9: 315–332.

[16] Mani MS (1964) The ecology of plant galls. Junk: The Hague. 604 p.

[17] Mani MS (1992) Introduction to cecidology. In: Shorthouse JD, Rohfritsch O, editors. Biology of insect-induced galls. Oxford: Oxford University Press. 3–7.

[18] Carneiro MAA, Branco CSA, Braga CED, Almada ED, Costa MBM, et al (2009) Are gall midge species (Diptera, Cecidomyiidae) host-plant specialists? Rev Bras Entomol 53: 365–378.

[19] Hartley SE (1998) The chemical composition of plant galls: are levels of nutrients and secondary compounds controlled by the gall-former? Oecologia 113: 492–501. 28308028 10.1007/s004420050401

[20] Inbar M, Izhaki I, Koplovich A, Lupo I, Silanikove N, et al (2010) Why do many galls have conspicuous colors? A new hypothesis. Arthropod Plant Interact 4: 1–6.

[21] Price PW (1991) The plant vigor hypotheses and herbivore attack. Oikos 62: 244–251.

[22] Espírito-Santo MM, Neves FS, Andrade-Neto FR, Fernandes GW (2007) Plant architecture and meristem dynamics as the mechanism determining the diversity of gall-inducing insects. Oecologia 153: 353–364. 17453251 10.1007/s00442-007-0737-8

[23] Cornelissen TG, Fernandes GW, Vasconcellos-Neto J (2008) Size does matter: Variation in herbivory between and within plants and the vigor hypothesis. Oikos 117: 1121–1130.

[24] Santos JC, Silveira FAO, Fernandes GW (2008) Long term oviposition preference and larval performance of *Schizomyia macrocapillata* (Diptera: Cecidomyiidae) on larger shoots of its host plant *Bauhinia brevipes* (Fabaceae). Evol Ecol 22: 123–137.

[25] Santos JC, Fernandes GW (2010) Mediation of herbivore attack and induced resistance by plant vigor and ontogeny. Acta Oecol 36: 617–625.

[26] Yukawa J (2000) Synchronization of gallers with host plant phenology. Popul Ecol 42: 105–113.

[27] Yukawa J, Akimoto K (2006) Influence of synchronization between adult emergence and host plant phenology on the population density of *Pseudasphondylia neolitseae* (Diptera: Cecidomyiidae) inducing leaf galls on *Neolitsea sericea* (Lauraceae). Popul Ecol 48: 13–21.

[28] Espírito-Santo MM, Fernandes GW (2002) Host plant effects on the development and survivorship of the galling insect *Neopelma baccharidis* (Homoptera: Psyllidae). Austral Ecol 27: 249–257.

[29] Lara DP, Oliveira AL, Azevedo IFP, Xavier MF, Silveira SAO, et al (2008) Relationship between host plant architecture and gall abundance and survival. Rev Bras Entomol 52: 78–81.

[30] Borchert R, Rivera G, Hagnauer W (2002) Modification of vegetative phenology in a tropical semi-deciduous forest by abnormal drought and rain, Biotropica. 34: 27–39.

[31] Sloan SA, Zimmerman JK, Sabat AB (2006) Phenology of *Plumeria alba* and its herbivores in a tropical dry forest. Biotropica 39: 195–201.

[32] Nunes YRF, Luz GR, Braga LL (2012) Phenology of tree species populations in Tropical Dry Forests of Southeastern Brazil. In: Zhang X., editor. Phenology and climate change. InTech, doi: 10.5772/34289, 125–142.

[33] Espírito-Santo MM, Madeira BG, Neves FS, Faria ML, Fagundes M, et al (2003) Sexual differences in reproductive phenology and their consequences for the demography of *Baccharis dracunculifoliae* (Asteraceae), a dioecious tropical shrub. Ann Bot 91: 13–19. 12495915 10.1093/aob/mcg001 PMC4240346

[34] Hesse E, Pannell JR (2011) Sexual dimorphism in a dioecious population of the wind-pollinated herb *Mercurialis annua*: the interactive effects of resource availability and competition. Ann Bot 107: 1039–1046. 21385775 10.1093/aob/mcr046 PMC3080628

[35] Munguia-Rosas A, Ollerton JEFF, Parra-Tabla V (2011) Phenotypic selection on flowering phenology and size in two dioecious plant species with different pollen vectors. Plant Spec Biol 26: 205–212.

[36] Ågren J (1988) Sexual differences in biomass and nutrient allocation in the dioecious *Rubus chamaemorus.* . Ecology 69: 962–973.

[37] Krischilk VA, Denno RF (1990) Patterns of growth, reproduction, defense, and herbivory in the dioecious shrub *Baccharis halimifolia* (Compositae). Oecologia 83: 182–190. 22160109 10.1007/BF00317750

[38] Delph LF, Lu Y, Jayne LD (1993) Patterns of resource-allocation in a dioecious *Carex* (Cyperaceae). Am J Bot 80: 607–615.

[39] Bullock SH, Bawa KS (1981) Sexual dimorphism and the annual flowering pattern in *Jacaratia dolichaula* (D. Smith) Woodson (Caricaceae) in a Costa Rican rain forest. Ecology 62: 1494–1504.

[40] Barroso GM (1976) Compositae-subtribo Baccharidinae-Hoffman: Estudo das espécies ocorrentes no Brasil. Rodriguesia 40: 7–273.

[41] Burckhardt D, Espírito-Santo MM, Fernandes GW, Malenovský I (2004) Gall-inducing jumping plant-lice of the Neotropical genus *Baccharopelma* (Hemiptera, Psylloidea) associated with *Baccharis* (Asteraceae). J Nat Hist 38: 2051–2071.

[42] Ferrari JM (1977) Vegetação do Campus da UFMG. Oréades 6: 3–5.

[43] Araújo AM, Fernandes GW, Bedê LC (1995) Influência do sexo e fenologia de *Baccharis dracunculifolia* DC. (Asteraceae) sobre insetos herbívoros. Rev Bras Entomol 39: 347–353.

[44] Marques AR, Fernandes GW, Reis IA, Assunção RA (2002) Distribution of adult male and female *Baccharis concinna* (Asteraceae) in the ruprestrian fields of Serra do Cipó, Brazil. Plant Biol 4: 94–103.

[45] Madeira JA, Fernandes GW (1999) Reproductive phenology of sympatric species of *Chamaecrista* (Leguminosae) in Serra do Cipó, Brazil. J Trop Ecol 15: 463–479.

[46] Crawley MJ (2002) Statistical computing: An introduction to data analysis using S-Plus. London: John Wiley & Sons. 772p.

[47] R Development Core Team (2010) R: A language and environment for statistical computing, Version 2.11.0., Vienna, Austria. Available in http://www.r-project.org.

[48] Espírito-Santo MM, Fernandes GW, Allain LR, Reis TRF (1999) Tannins in *Baccharis dracunculifolia* (Asteraceae): effects of seasonality, water availability and plant sex. Acta Bot Bras 13: 167–174.

[49] Araújo APA, Carneiro MAA, Fernandes GW (2003) Efeitos do sexo, do vigor e do tamanho da planta hospedeira sobre a distribuição de insetos indutores de galhas em *Baccharis pseudomyriocephala* Teodoro (Asteraceae). Rev Bras Entomol 47: 483–490.

[50] Matsushita M, Nakagawa M, Tomaru N (2011) Sexual differences in year-to-year flowering trends in the dioecious multi-stemmed shrub *Lindera triloba*: effects of light and clonal integration. J Ecol 99: 1520–1530.

[51] Rocheleau AF, Houle G (2001) Different cost of reproduction for the males and females of the rare dioecious shrub *Corema conradii* (Empetraceae). Am J Bot 88: 659–666. 11302852

[52] Antos JA, Allen GA (1999) Patterns of reproductive effort in male and female shrubs of *Oemleria cerasiformis*: a 6-year study. J Ecol 87: 77–84.

[53] Gomes V, Fernandes GW (2002) Germinação de aquênios de *Baccharis dracunculifolia* D.C.(Asteraceae). Acta Bot Bras 16: 421–427.

[54] Janzen DH (1967) Synchronization of sexual reproduction of trees within the dry season in Central America. Evolution 21: 620–637. 28563685 10.1111/j.1558-5646.1967.tb03416.x

[55] Madeira BG, Cornelissen TG, Faria ML, Fernandes GW (1997) Insect herbivore preference for sex and modules in *Baccharis concinna* (Asteraceae). In: Raman A, editor. Ecology and evolution of plant-feeding insects in natural and man-made environments. New Delhi: International Scientific Publications. 135–145.

[56] Detoni ML, Vasconcelos EG, Maia ACRG, Gusmão MAN, Isaías RMS, et al (2011) Protein content and electrophoretic profile of insect galls on susceptible and resistant host plants of *Bauhinia brevipes* Vogel (Fabaceae). Aust J Bot 59: 509–514.

